# Characterization of factors underlying the metabolic shifts in developing kernels of colored maize

**DOI:** 10.1038/srep35479

**Published:** 2016-10-14

**Authors:** Chaoyang Hu, Quanlin Li, Xuefang Shen, Sheng Quan, Hong Lin, Lei Duan, Yifa Wang, Qian Luo, Guorun Qu, Qing Han, Yuan Lu, Dabing Zhang, Zheng Yuan, Jianxin Shi

**Affiliations:** 1 State Key Laboratory of Hybrid Rice, Shanghai Jiao Tong University–University of Adelaide Joint Centre for Agriculture and Health, School of Life Sciences and Biotechnology, Shanghai Jiao Tong University, Shanghai 200240, China; 2 Shanghai Academy of Agricultural Sciences, Shanghai 201403, China; 3 Plant Genomics Center, School of Agriculture, Food and Wine, University of Adelaide, Waite Campus, Urrbrae, South Australia 5064, Australia; 4 Key Laboratory of Crop Marker-Assisted Breeding of Huaian Municipality, Jiangsu Collaborative Innovation Center of Regional Modern Agriculture and Environmental Protection, Huaian 223300, China

## Abstract

Elucidation of the metabolic pathways determining pigmentation and their underlying regulatory mechanisms in maize kernels is of high importance in attempts to improve the nutritional composition of our food. In this study, we compared dynamics in the transcriptome and metabolome between colored SW93 and white SW48 by integrating RNA-Seq and non-targeted metabolomics. Our data revealed that expression of enzyme coding genes and levels of primary metabolites decreased gradually from 11 to 21 DAP, corresponding well with the physiological change of developing maize kernels from differentiation through reserve accumulation to maturation, which was cultivar independent. A remarkable up-regulation of anthocyanin and phlobaphene pathway distinguished SW93 from SW48, in which anthocyanin regulating transcriptional factors (*R1* and *C1*), enzyme encoding genes involved in both pathways and corresponding metabolic intermediates were up-regulated concurrently in SW93 but not in SW48. The shift from the shikimate pathway of primary metabolism to the flavonoid pathway of secondary metabolism, however, appears to be under posttranscriptional regulation. This study revealed the link between primary metabolism and kernel coloration, which facilitate further study to explore fundamental questions regarding the evolution of seed metabolic capabilities as well as their potential applications in maize improvement regarding both staple and functional foods.

Maize (*Zea mays* ssp. *mays* L.) is of global significance not only as a food, feed and a source of diverse industrial products, but also as a model system with tremendous genetic diversity for general plant biology studies as well as for specific biological phenomena such as heterosis and gene transposition^1^,^2^. Its storage organs, the kernels, contain essential chemical components necessary for seed germination and seedling growth, and essential nutrients for human food and livestock feed. Similar to other cereals such as rice, the value of the maize kernel is mainly determined by its endosperm^3^. Therefore, the development of maize endosperm has attracted special attention in the past decades. Maize kernel development includes endosperm development and embryo development, two separate but associated phases. The three endosperm development stages are distinct but overlapped. The early stage initiates from 1 to 4 DAP, containing double fertilization, syncytium formation and cellularization; the differentiation stage lasts from 4 to 20 DAP, including cell type formation, mitotic cell proliferation, endoreduplication and reserve accumulation; while the maturation stage begins from around 16 to about 60 DAP, comprising programmed cell death, dormancy and desiccation^3^. Along the endosperm development, the maize embryo develops from a pro-embryo (5 DAP) into a well differenced embryo (15 DAP) in several distinguishable but continuous stages, including pro-embryo formation and initiation (7 DAP), rapid enlargement of the scutellum (9–12 DAP), and embryo maturation (13–15 DAP)^4^,^5^. The timing of kernel development, however, depends largely on genetic background and environmental conditions^5^.

Previous studies reveal that the physiological and morphological changes along maize kernel development result mainly from developmental cue-regulated gene expression and the functions of the proteins that they encode at specific developmental stages. At the transcriptional level, the gene expression profiles of developing maize kernels are clustered into three distinct groups: 2–8 DAP, 10–14 DAP and 16–24 DAP^6^, those of developing maize endosperm are classified into 12–18 DAP and 20–24 DAP groups, while the differences of transcriptomes of maize embryos at 16–24 DAP are much smaller than those of seeds and endosperms^6^. Another transcriptomic study revealed remarkable changes in the pattern of certain functional genes in endosperm transcriptome during storage accumulation stage (10, 14 and 21 DAP)^7^. In addition, extensive proteomic studies on maize kernel development revealed that the abundance of proteins that are involved in cell division, respiration, detoxification and energy production is higher at early stages, while that of proteins related to storage protein synthesis, protein folding and glycolysis is higher at relative late stages^8^,^9^,^10^,^11^. These findings described above indicated that maize kernel development is regulated at least at both transcriptional and post-transcriptional levels.

Metabolites, the final products of the cellular regulatory process^12^, represent the building blocks for macromolecules and are also an essential component of cellular energy. Metabolomic analysis of the kinetic metabolic patterns of developing seeds in plants such as Arabidopsis^13^, tomato^14^ and rice^15^, have provided important molecular and biochemical insight into our understanding of seed development. In maize, a pioneer study reported that the contents of dry weight, total nitrogen, protein, fat and DNA in a kernel increase gradually along seed development while those of total amino acids and soluble nucleotides first increase to the maximum value at 28 DAP and decrease afterwards^16^. Several recent excellent works concerning the natural variation of mature maize kernel metabolomes also provide useful information about the chemical diversity in maize kernels^17^,^18^,^19^,^20^, however, a detailed comprehensive analysis of the dynamic metabolic changes in developing kernels has not been carried out. Determination of the molecular and biochemical mechanisms governing kernel development in maize is of significance not only for basic research on maize development but also for maize improvement via metabolic engineering.

Color development is an important event of maize kernel development. During their adaptation to new growth environments and particularly due to artificial selection by humans, maize kernels lost their colors and as a consequence globally cultivated maize are mainly yellow kernels^21^. Recently, there is increasing demand for dark-colored maize kernels because of the rising awareness of the nutritional importance of pigments in the prevention of chronic diseases^22^,^23^,^24^. Therefore, considerable attentions have been drawn back to ancient flavonoid-rich landraces with attempts to understand the color formation in a range of crops including maize kernels^21^,^25^. It is well known that flavonoids, mainly anthocyanins and specifically phlobaphenes, account for the colorful pigments in maize kernels^21^,^26^,^27^. Anthocyanin compounds are responsible for blue, purple and red hues, while phlobaphenes contribute mainly to red and purple hues^21^,^26^,^27^. The anthocyanin pathway in maize is transcriptionally regulated by a ternary complex of transcription factors, including the MYB factor C1 (colorless 1) or PL1 (purple leaf 1), the bHLH factor R1 (red color 1) or B1 (booster 1), and the WD40 factor PAC1 (pale aleurone color 1)^28^,^29^,^30^. Although each transcription factor appears to be regulated independently, the expression of all three is required for anthocyanin pigmentation^21^,^31^. By contrast, the biosynthesis of phlobaphene is controlled by the MYB member P1 (Pericarp color1) in the absence of R1 or B1^27^,^32^,^33^. Transcriptionally, these transcriptional factors function via regulating three common flavonoid biosynthetic structural genes, *chalcone synthase (CHS*), *chalcone isomerase (CHI*) and *dihydroflavonol 4-reductase (DFR*), to produce precursors of various anthocyanins and phlobaphenes. In addition, because of the remarkably uncompact structure of the promoters of *R1*^34^,^35^,^36^, *B1*^35^,^37^, and *P1*^38^,^39^ genes, epigenetics also plays important roles in the regulation of the complicated spatial and temporal pigmentation in maize. Nevertheless, most current understanding of maize kernel pigmentation has been obtained from conventional genetic studies using mutants of the above structural and regulatory genes. To date a systematic investigation of the molecular and biochemical mechanisms underlying the complex pigmentation in maize kernels is still missing.

In this study, taking the advantage of the system biology approach, we comparatively investigated the dynamic transcriptomic and metabolomic profiles of developing maize kernel in two different maize cultivars, SW48 and SW93. By interactively comparing metabolomic and transcriptomic data between SW48 and SW93, we were able to draw a comprehensive picture of events underpinning the maize kernel development corresponding well with the respective physiological and phenotypic changes. The transcriptional and metabolic changes was in close agreement with the onset of pigmentation in SW93 maize kernels, and the comparison of the two lines allowed considerable insight into the regulatory mechanisms underlying pigmentation in maize kernels

## Results

### Changes in Pigmentation Pattern

**Figure 1:**
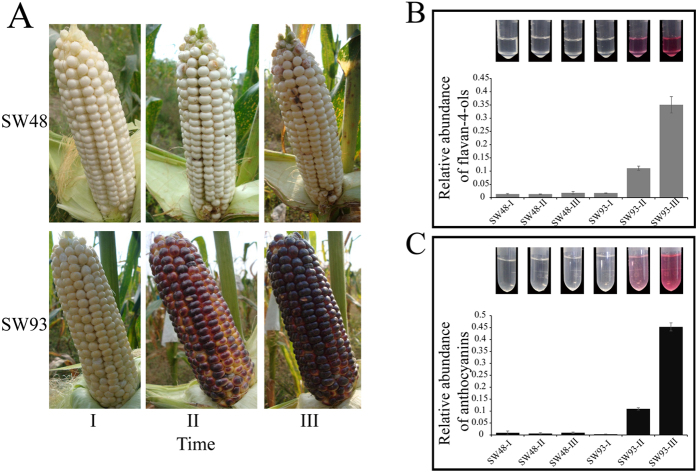
The development and coloration of maize kernels. (**A**) Phenotypes of developing maize ears of SW48 and SW93 along the development in the field; (**B**) The accumulation of phlobaphene precursors in SW93 kernels along the development; (**C**) The accumulation of anthocyanins in SW93 kernels along the development.

At 11 DAP, the maize kernel of SW48 and SW93 were both white. At 16 and 21 DAP, the kernel color of SW93 changed from light purple or red to dark purple while that of SW48 remained white (Fig. 1A). Notably, across tested developmental stages, flavan-4-ols (the precursor of the phlobaphene) and total anthocyanins accumulated in SW93 but not in SW48 at 16 and 21 DAP (Fig. 1B), respectively, corresponding well with the kernel coloration patterns (Fig. 1A), indicating that two flavonoids branches (phlobaphene and anthocyanin pathways) are associated with the kernel coloration in SW93.

### Overall Kinetics of Transcriptomic Changes

**Figure 2:**
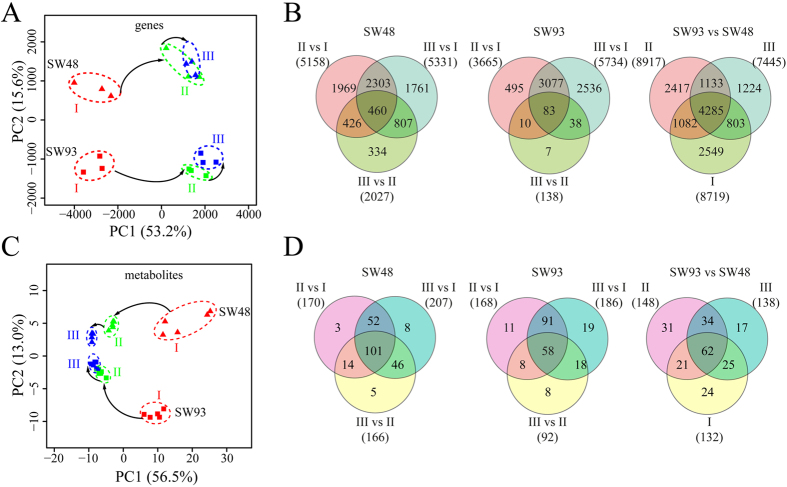
The overview of the transcriptomic and metabolomic changes in developing maize kernels. (**A**,**C**) Principle component analysis of detected transcriptomic data and metabolomic data, respectively. Red, green and blue colors represent samples at I, II and III, respectively. Box and triangle denote samples of SW93 and SW48, respectively. I, II and III refer to 11, 16 and 21 DAP, respectively. (**B**,**D**) Venn diagrams of differentially expressed genes (DEGs), and differential metabolites (DAMs), of maize kernel at different time points of the same cultivar and at the same time point of different cultivars. I, II and III refer to 11, 16 and 21 DAP, respectively.

Transcriptomic analysis based on RNA-seq revealed that about 51,000 transcripts (accounting for 46% of all maize transcripts) were expressed in developing maize kernels (Supplementary Table S1). Principle component (PC) analysis on all those expressed transcripts indicated that PC 1, explaining 53.2% of the total variance, separated samples at 11 DAP from those at 16 DAP and 21 DAP (Fig. 2A), and demonstrated that the transcriptome of maize kernel at 11 DAP was significantly different from those at 16 and 21 DAP in both cultivars. The fact that samples at 16 and 21 DAP were closely grouped and almost indistinguishable in both cultivars (Fig. 2A) indicated that in both cultivars the transcriptomes of developing kernels at these two stages were highly similar. PC 2, accounting for 15.6% of the total variance, separated samples of SW48 from those of SW93 (Fig. 2A), which demonstrated distinct kernel transcriptomes between SW48 and SW93 at all three tested time points. Notably, the scores of samples at 11 DAP and those at 16 and 21 DAP were distributed on the left and right sides of the plot, respectively (Fig. 2A). When PCA was applied to all detected 1867 enzyme coding genes (ECG) based on Kyoto Encyclopedia of Genes and Genomes (KEGG) database (http://www.kegg.jp/) and Plant Metabolic Pathway Database (http://www.plantcyc.org/), similar separation patterns of samples at 11 DAP from those at 16 and 21 DAP was also observed (Supplementary Fig. S1). However, the samples at 11 DAP and those at 16 and 21 DAP were distributed on the right and left sides of the plot, respectively (Supplementary Fig. S1), notably different from that of all expressed genes (Fig. 2A).

In SW48, as compared with samples of 11 DAP, the numbers of differentially expressed genes (DEG) in samples of 16 DAP and 21 DAP were 5,158 (3,166 up and 1,992 down) and 5,331 (3,557 up and 1,774 down), respectively, and as compared with samples of 16 DAP, the number of DEG in samples of 21 DAP was 2,027 (1,322 up and 705 down) (Fig. 2B). Similarly, in SW93, as compared with samples of 11 DAP, the numbers of DEG in samples of 16 DAP and 21 DAP were 3,665 (2,547 up and 1,118 down) and 5,734 (3,921 up and 1,813 down), respectively, and as compared with samples of 16 DAP, the number of DEG in sample of 21 DAP was 138 (124 up and 14 down) (Fig. 2B). These data indicated that the numbers of developmental associated DEGs increased similarly in both cultivars.

### Overall Kinetics of Metabolomic Changes

Metabolomic analysis based on UHPLC-MS/MS and GC-MS identified a total of 247 metabolites with known structures in developing maize kernels (Supplementary Table S2). PCA on these metabolites uncovered that PC1, explaining 56.5% of total variance, separated samples at 11 DAP from those at 16 and 21 DAP, and PC2, accounting for 13.0% of left variance, separated SW93 from SW48 (Fig. 2C). Thus, metabolome of kernels at 11 DAP was significantly different from those at 16 and 21 DAP in both cultivars, and metabolomes of SW48 at 11, 16 and 21 DAP were distinct from those of SW93. Remarkably, the metabolite scores of samples at 11 DAP and those at 16 and 21 DAP were distributed on the right and left sides of the plot, respectively, which is similar to the plot of ECGs (Supplementary Fig. S1). Therefore, unless stated, gene expression analysis targets ECGs.

In SW48, as compared with samples of 11 DAP, the numbers of differentially altered metabolites (DAM) (P-value ≤ 0.05) in samples of 16 DAP and 21 DAP were 170 and 207, respectively, and as compared with samples of 16 DAP, the number of DAM in sample of 21 DAP was 116 (Fig. 2B). Similarly, in SW93, as compared with samples of 11 DAP, the numbers of DAM in samples of 16 DAP and 21 DAP were 168 and 186, respectively, and as compared with samples at 16 DAP, the number of DEG in sample of 21 DAP was 92 (Fig. 2D). This revealed metabolomic kinetics is similar to that of transcriptomic kinetics (Fig. 2B).

### Transcriptomic and Metabolomic Variations between SW48 and SW93

There were remarkable transcriptional and metabolomic variations between SW48 and SW93 across kernel development. As compared with SW48, the numbers of DEG and DAM in SW93 samples at 11 DAP were 8,719 and 132, respectively, those at 16 DAP were 8,917 and 148, respectively, and those at 21 DAP were 7,445 and 138, respectively (Fig. 2B and Fig. 2D).

**Figure 3:**
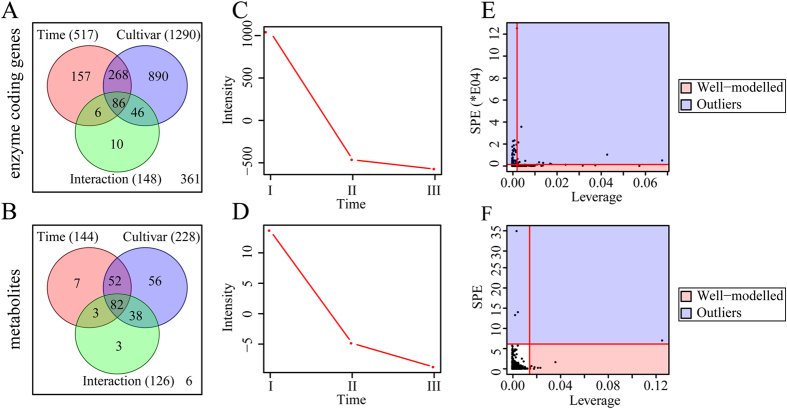
Result of two-way ANOVA and ASCA. (**A**,**B**) Venn diagram of enzyme coding genes and metabolites whose levels were affected by time, cultivar or time-cultivar interaction; (**C**,**D**) Major pattern of enzyme coding genes and metabolites associated with time. I, II and III refer to 11, 16 and 21 DAP, respectively; (**E**,**F**) ASCA selection of important enzyme coding genes and metabolites associated with time by Leverage/SPE analysis. These analysis was performed in MetaboAnalyst website http://wwwmetabolanalyst.ca website^63^.

Because PCA is not able to distinguish the exact contribution of each variable to the observed variation, therefore, two-way ANOVA and ASCA were conducted to decompose both transcriptional and metabolic variations derived from time, cultivar and their interaction (Fig. 3). ANOVA revealed that the numbers of DEG affected significantly by time, cultivar and their interaction were 517, 1,290, and 148, respectively (Fig. 3A, Supplementary Table S3), while those of DAM were 144, 228 and 126, respectively (Fig. 3B, Supplementary Table S4). In addition, ASCA revealed that 55.5%, 10.0% and 14.2% of observed variations of ECG (Supplementary Fig. S2), and 55.5%, 9.7%, and 12.5% of observed variations of metabolite levels (Supplementary Fig. S3), could be explained by time, cultivar and their interaction, respectively. Time score plots based on PC1 of the corresponding submodels also showed similar transcriptional and metabolomic changes, in both cases, the scores gradually decreased from 11 DAP to 21 DAP (Fig. 3C,D), indicating a conserved and coordinated reduction in the transcriptional and metabolic activities across maize kernel development. Furthermore, Leverage/SPE analysis 40,41 revealed that the numbers of identified ECGs responsible for observed variations derived from time, cultivar, and their interaction were 56, 32, and 25, respectively (Fig. 3E; Supplementary Table S5), and those for DAMs were eight, nine, and seven, respectively (Fig. 3F; Supplementary Table S6). Further investigating deeply into those responsible ECGs and DAMs uncovered that each variable has specific ECGs and/or DAMs (Supplementary Tables S5 and S6). For example, the 56 ECGs identified as time variable were associated mainly with energy metabolism; the 32 ECGs identified as being cultivar variable were involved in 21 metabolic pathways; the 25 ECGs identified as being dependent on the interaction variable participated in diverse metabolic pathways including the flavonoid biosynthetic pathway (Supplementary Table S5). Similarly, the eight DAMs identified as being time variable were involved in the metabolisms of amino acids, lipids and auxin; the nine DAMs identified as being cultivar variable participated in the metabolisms of amino acids, carbohydrates, cofactors and phenylpropanoids; while the seven DAMs identified as being dependent on the interaction variable were associated with the metabolisms of cofactors, nucleotides, lipids and flavonoids (Supplementary Table S6).

### Conserved and Divergent Developmental Induced Transcriptomic and Metabolomic Alterations between SW48 and SW93

GO analysis on DEGs revealed that these two cultivars differed in GO terms associated with signaling, metabolic, and stress responsive activities at 11 DAP, in starch metabolic, stress responsive and oxidative-reductive activities at 16 DAP, and in metabolic and stress responsive activities at 21 DAP (Supplementary Table S7). Notably, GO terms related to metabolism, such as “anthocyanin-containing compound biosynthetic process” and “flavonoid biosynthetic process” were markedly enriched at 21 DAP in SW93.

**Figure 4:**
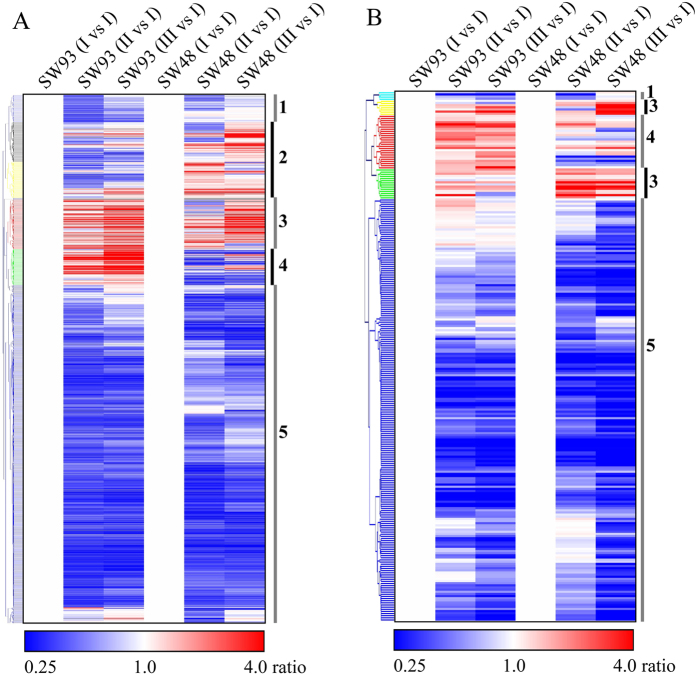
Hierarchical clustering analysis of the transcriptomic and metabolomic data. (**A**) enzyme coding genes; (**B**) metabolites. Red color indicate the abundance of enzyme coding gene or metabolite tends to be up-regulated compared to that at I of the same cultivar and blue down-regulated see ratio color key. 1 and 5, cluster 1 and cluster 5: down-regulated in both SW93 and SW48; 2, cluster 2: down-regulated in SW93, but up-regulated in SW48; 3, cluster 3: up-regulated in both SW93 and SW48; 4, cluster 4: up-regulated in SW93, but down-regulated in SW48.

Hierarchical clustering analysis on 1867 differentially expressed ECGs showed that 72% DCGs (1,348/1,867) were down-regulated at 16 and/or 21 DAP as compared with those at 11 DAP in both cultivars, although the degree of the decrease varied among genes (Fig. 4A, cluster 1). This result is consistent with the ASCA time score plot (Fig. 3C) and these downregulated genes covered almost all the detected metabolic pathways (Supplementary Table S8). The expression of 246 ECGs were upregulated in SW48 but downregulated or unaltered in SW93 at 16 and/or 21 DAP (Fig. 4A, cluster 2) and 80% of them were involved in primary metabolism while the other 20% participated in secondary metabolism (Supplementary Table S8). The expressions of 170 ECGs were upregulated in both SW93 and SW48 at 16 and/or 21 DAP (Fig. 4A, cluster 3), all these ECGs were involved in either primary or secondary metabolism. The expression of 91 ECGs were upregulated in SW93 but were downregulated or not significantly changed in SW48 at 16 and/or 21 DAP (Fig. 4A, cluster 4), ECGs involved in flavonoids metabolism were found among them (cluster 4).

Hierarchical clustering analysis on identified metabolites showed that abundances of 81.4% (201/247) of the identified metabolites decreased at 16 and/or 21 DAP as compared with those at 11 DAP in both kernels (Fig. 4B, cluster 1), which was similar to the time score plot from ASCA (Fig. 3D). The levels of 21 metabolites increased at 16 and/or 21 DAP in both SW93 and SW48, but with relative higher levels in SW48 than those in SW93 (Fig. 4B, cluster 3); these metabolites were involved mainly in primary metabolism (Supplementary Table S9). The levels of 11 metabolites increased at 16 and 21 DAP significantly in SW93 while slightly changed at 16 and/or 21 in SW48 (Fig. 4B, cluster 4); among them, naringenin is a secondary metabolite.

### The Flavonoid Pathway Is Activated in Developing SW93 Kernels

**Figure 5:**
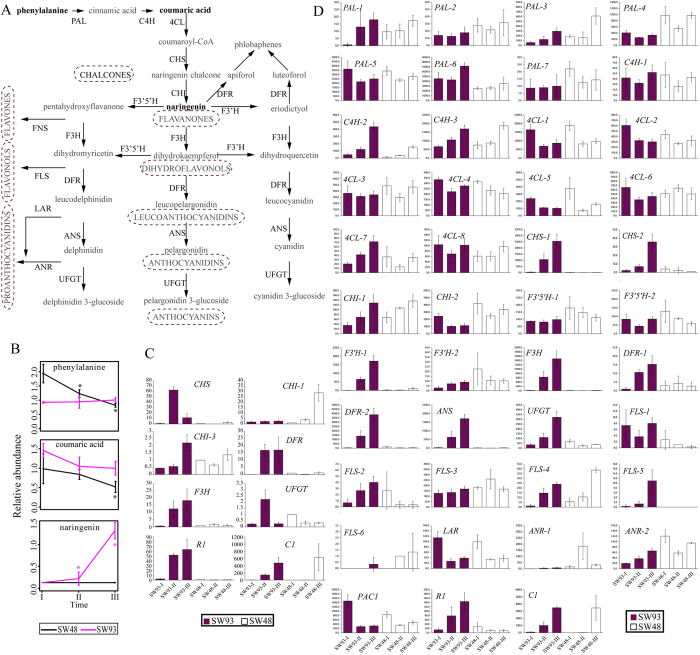
Integration of gene expression and metabolite changes of flavonoids pathway. (**A**) Simplified map of anthocyanin biosynthesis pathway modified from Petroni *et al.*^21^. The font style of the identified metabolites were set to be bold. PAL, phenylalanine ammonia lyase; C4H, cinamic acid 4-hydroxylase; 4CL, 4-coumarate CoA ligase; CHS, chalcone synthase; CHI, chalcone isomerase; DFR, dihydroflavonol reductase; F3′H, flavanone 3′-hydroxylase; F3H, flavanone 3-hydroxylase; F3′5′H, flavonoid 3′,5′-hydroxylase; ANS, anthocyanidin synthase; UFGT, UDP-flavonoid glucosyl transferase; FNS, flavone synthase; FLS, flavonol synthase; LAR, leucoanthocyanidin reductase; ANR, anthocyanidin reductase. B, Changes in metabolite levels in maize kernels along the development. *denotes the metabolite level at II (16 DAP) or III (21 DAP) was significantly different from that at I (11 DAP) of the same cultivar. Black and purple stars denote SW48 and SW93, respectively. C, Changes in expression levels of genes involved in flavonoids pathway as revealed by qRT-PCR. *C1*, *colored aleurone 1* or *colorless1*; *R1*, *red color 1*. The y-axis represents the relative abundance of each gene. The mean expression level of each gene at I of SW48 was denoted as 1. CHS: GRMZM2G422750 and GRMZM2G151227; CHI-1: GRMZM2G119186; CHI-2: GRMZM2G155329; DFR: GRMZM2G026930 and GRMZM2G013726; F3H: GRMZM2G062396. C1: GRMZM2G005066; R1: GRMZM5G822829. D, Changes in expression levels of genes involved in flavonoids pathway as revealed by RNA-Seq. The y-axis represents the counts of genes in SW93 and SW48 by RNA-Seq. The detailed expression profile of each gene was shown in Supplementary Table S8.

Increases in the contents of total anthocyanins and flavan-4-ol in SW93 kernels at 16 and 21 DAP (Fig. 1) led us to focus on the transcriptional and metabolic changes in flavonoid pathway (Fig. 5). At the transcriptional level, RNA-Seq data found that neither anthocyanin regulator *B1* (GRMZM2G172795), *PL1* (GRMZM2G701063) nor phlobaphene regulator *P1* (GRMZM2G084799 and GRMZM2G057027) was detected in kernels of SW93 and SW48 across kernel development (Supplementary Table S1). However, the expression levels of anthocyanin regulators *R1* (GRMZM5G822829) and *C1* (GRMZM2G005066) were remarkably increased in SW93 but not in SW48 across kernel development (with the exception of *C1* at 21 DAP) (Fig. 5D). The expression level of the third anthocyanin regulator, a WD40 protein, PAC1 (GRMZM2G058432), was expressed highly in both kernels at 11 DAP, which declined similarly at 16 and 21 DAP. Notably, two other transcription factors, AC194965.4_FG004 (GATA family) and GRMZM2G065374 (bHLH family), showed similar expression patterns to *R1* in SW93 (Supplementary Fig. S4). In addition, the expression levels of two structural genes commonly involved in both anthocyanin and phlobaphene pathways namely *CHS* and *DFR* were upregulated in SW93 across kernel development, including two *CHS* (GRMZM2G151227 and GRMZM2G422750) and two *DFR* (GRMZM2G013726 and GRMZM2G026930) (Fig. 5D). Notably, one *CHI* transcript (GRMZM2G119186) increased across kernel development in both kernels (Fig. 5D). Furthermore, the levels of transcripts of *ANS (anthocyanidin synthase*) (GRMZM2G345717), *F3H (flavanone 3-hydroxylase*) (GRMZM2G062396), and *UFGT (UDP-flavonoid glucosyl transferase*) (GRMZM2G165390), all displayed similar expression patterns as *CHS* and *DFR* across kernel development. These findings indicated a concurrent activation of both anthocyanin and phlobaphene pathways in SW93. The activation of phlobaphene pathway in SW93 was also evidenced by the increased expression of one transcript of *F3′H (flavanone 3′-hydroxylase*) (GRMZM2G025832), a direct target of *P1*^42^, in SW93 but not in SW48 (Fig. 5D). Moreover, RNA-Seq data demonstrated that although the expression levels of transcripts of upstream genes of seven *PAL (phenylalanine ammonia-lyase*), three *C4H (cinnamate 4-hydroxylase*), and eight *4CL (cinanic acid 4-hydroxylase*) fluctuated across kernel development in SW93, their patterns were quite similar to those in SW48 (Fig. 5D). This divergence was also confirmed by the observed differential expression levels of transcripts of genes downstream of *FLS (flavonol synthase*) (Fig. 5D). The expression pattern changes of several genes across kernel development from 11 to 21 DAP were confirmed by qualitative RT-PCR (Fig. 5C).

Although non-targeted UPLC-MS-MS only identified three metabolites in the phenylalanine pathway, they all exhibited distinct kinetic patterns across kernel development between SW93 and SW48. The abundance of phenylalanine declined continuously in SW48 whilst it was unaltered in SW93 across kernel development, the level of coumaric acid in SW93 was always significantly higher than that in SW48 across kernel development, while the level of naringenin, was remarkably increased across kernel development in SW93 whilst being kept at a basal level in SW48 (Fig. 5B). Altogether, these findings strongly indicated the developmental clue redirected metabolic shift to flavonoids pathway in SW93, leading to colored kernels.

### Other Genotype Specific Changes

#### Glycolysis

RNA-Seq data revealed that the expression levels of most of the 54 genes encoding 12 enzymes involved in glycolysis were down-regulated across kernel development in both kernels (Supplementary Fig. S5B), while those of transcripts coding for a hexokinase (EC 2.7.1.1), a 6-phosphofructokinase (EC 2.7.1.11) and a fructose-bisphosphate aldolase (EC 4.1.2 13) were significantly up-regulated only in SW93 at 16 and 21 DAP (Supplementary Fig. S5B). On the other hand, metabolomic analysis showed that levels of three glycolytic intermediates (glucose, glucose-6P and fructose-6P) decreased and that of phosphoenolpyruvate (PEP) increased in both cultivars during development (Supplementary Fig. S5A). Notably, different from that in SW48, the level of pyruvate, the end-product of glycolysis and the indispensable precursor of the tricarboxylic acid (TCA) cycle remained constant across kernel development in SW93 (Supplementary Fig. S5A).

#### The TCA cycle

RNA-Seq data revealed that the expression levels of almost all TCA genes were significantly down-regulated at 16 and 21 DAP in both cultivars (Supplementary Fig. S6C). Notably, those of the transcripts coding for citrate oxaloacetate-lyase (EC 2.3.3.1) and for isocitrate dehydrogenase (EC 1.1.1.42) were significantly up-regulated in SW93 (Supplementary Fig.S6C). Six metabolites involved in TCA cycle were identified and the levels of them all decreased in both SW93 and SW48 (Supplementary Fig. S6A). It is worth noting however that the level of α-ketoglutarate in SW93 was consistently higher than that in SW48 across kernel development, and that of citrate at 21 DAP in SW93 was obviously higher than that in SW48, which is in close agreement with above-mentioned upregulation of the genes encoding for citrate oxaloacetate lyase and isocitrate dehydrogenase.

#### Other differentially altered pathways

Other pathways were differentially affected between the genotypes but these changes were mainly apparent at the metabolite level. In pentose phosphate pathway, the level of glucose, the precursor of this pathway was stable, while the levels of the two pentose intermediates xylulose-5-phosphate and ribose-5-phosphate, were lower in SW93 kernel across kernel development (Supplementary Fig. S7). In purine metabolism, the levels of 2′3′-cyclic AMP, 2′3′-cyclic GMP, and 3′-AMP were significantly higher while that of adenosine was remarkably lower in SW93 than those in SW48 across kernel development (Supplementary Fig. S8). Notably, the change of the abundance of allantoin in two cultivars was opposite during development. In amino acid pathway, the level of tryptophan was stable in SW93, while the levels of proline and *allo*-threonine were notably higher in SW93 (Supplementary Fig. S9). In nicotinate pathway, the level of nicotinate was consistently lower and stable, while that of trigonelline was significantly higher in SW93 (Supplementary Fig. S10).

## Discussion

During seed development, various metabolites are synthesized and stored, making seeds chemical factories of stored nutrients. Understanding the complete and holistic knowledge of seed development events, particularly their reserve accumulation mechanisms, is therefore a primary prerequisite for guaranteeing the global food security via knowledge-based genetic improvement^43^. In this study, we comparatively characterized the dynamic transcriptome and metabolome of two maize kernels of different pigmentation during the reserve accumulation and maturation phases of seed development. Our results revealed many conserved transcriptomic and metabolomic events in those two maize cultivars, which are remarkably similar to reported observations in other dicot plants^13^,^14^. However, our data also uncovered a considerable molecular (transcriptional and metabolic) shift in colored maize kernels; potential regulatory aspects uncovered by this are discussed below in the context of the flavonoids biosynthesis.

### Transcriptomic and Metabolomic Kinetics of Maize Kernel Development

Based on both transcriptomic and metabolomic data obtained in this study, there was clear decline in gene expression and enzyme metabolic activity in maize kernels from 11 DAP till 21 DAP, corresponding to the maize seed developmental stages from reserve accumulation to maturation (Fig. 3C,D). This resulted in two developmental stage dependent transcriptomes and metabolomes, with those at 16 DAP being easily distinguishable from those at 11 DAP but only moderately distinguishable from those at 21 DAP (Figs 2 and 4). These molecular changes correspond well to the physiological and morphological changes occurring in parallel across kernel development. Kernels at 11 DAP were active in reserve accumulation, while those at 16 DAP were approaching maturation^3^, therefore, the active genes and resulting metabolites showed substantial decline when kernels developed from 11 DAP to 16 DAP and afterwards. The cultivar independent transcriptomic and metabolomic kinetics observed in both developing maize kernels suggested a highly conserved regulatory mechanism within maize seed development, which is quite similar to previous reports in rice^15^, tomato^14^,^44^, and Arabidopsis^13^. Notably, the genome wide expression patterns of all genes were somewhat divergent than those of ECGs (Fig. 2A and Supplementary Fig. S1), which might, at least partially, explain the imperfect match of metabolomic data to transcriptomic data^45^ when all detected DEGs as opposed to differentially expressed ECGs are compared. Previous proteomic and transcriptomic studies have already stressed the remarkable changes in maize kernels at reserve accumulation stages^6^,^7^. Thus, our findings appear to be conserved across a wide range of maize cultivars as well as in other crops, highlighting the value in identifying key genes for seed development even in those species whose seeds exhibit remarkable differences in size and storage components.

### The Metabolic Shifts in Developing Colored Maize Kernels

Understanding of the pathways of pigmentation and their regulation in kernels is of high commercial importance in maize and other related cereal crops. Considerable progresses has been made in defining and understanding the metabolic pathways leading to the colorful maize kernels, however, few studies have attempted to integrate changes in primary metabolism to coloration and as such our ability to influence maize kernel coloration is arguably not as great as it should be. While this study reveals that although the general kinetic trends in transcriptome and metabolome of both SW93 and SW48 were quite similar across kernel development, the transcriptional and metabolic changes in SW93 were clearly associated with the coloration in its kernels. The substantial transcriptional and metabolic shifts in SW93 occurred evidently at 16 DAP (Fig. 4), which coincided with the appearance of pigment in SW93 kernels at this stage (Fig. 1), indicating an irreversible shift to secondary metabolism in SW93, producing anthocyanins and precursors of phlobaphene, two important secondary metabolites, to color the maize kernels with colorful pigments. This result may also be explained by a likely existence of a transcription factor in SW93 that targets both primary and secondary metabolism as reported for tomato SlMYB12^24^ and its maize counterpart P1^27^. Under certain conditions, adequate supplies of precursors, energy and reducing power form the primary metabolism are required to meet demands of specialized secondary metabolism.

In plants, the shikimate pathway plays important roles in plant growth, development and response to environment by providing three aromatic amino acids (tryptophan, tryosine and phenylalanine) that are essential precursors for the secondary metabolism, with phenylalanine being the direct precursor for pigmentation in most plants^46^. Whilst no clear transcriptional activation of the whole shikimate pathway was observed in developing SW93 kernels, the level of a transcript (GRMZM2G125923) encoding an arogenate/prephenate dehydratase (EC 4.2.1.91) was only upregulated in SW93, which led to a constant phenylalanine level in SW93 cross kernel development, contrasting to the significant decline observed in SW48 (Supplementary Fig. S11). The lack of a coordinated response in the shikimate pathway is arguably unsurprising since plants, unlike the AroM complex of fungi in which a single penta-functional protein is responsible for shikimate biosynthesis, do not have a gene cluster for the shikimate pathway and indeed the gene expression patterns of the constituent enzymes are somewhat distinctive^47^. Interestingly, tryptophan displayed similar metabolic kinetics to those of phenylalanine without upregulation of corresponding biosynthetic genes such as anthranilate synthase (EC 4.1.3.27) and chorismate mutase (EC 5.4.99.5), suggesting that stable metabolic supplies for tryptophan and phenylalanine are likely maintained by shikimate pathway via as yet unknown post-transcriptional mechanisms.

The stable supply of phenylalanine in developing kernels of SW93 was likely derived directly from glycolysis and TCA cycle. As compared with SW48, the level of PEP was constantly lower in SW93. PEP is the important entry metabolite from glycolysis to shikimate pathway, both oxaloacetate from TCA and pyruvate from glycolysis are important precursors for PEP^48^. Notably, levels of oxaloacetate and pyruvate in SW93 were constant but those in SW48 kept decline along the kernel development. These results indicated a metabolic shift from central carbon metabolism to amino acid metabolism in developing SW93 kernels, which could also be evidenced by the observed higher levels of amino acids and their derivatives derived from PEP (tyramine), oxaloacetate (*allo*-threonine, isoleucine and 2-aminoadipate), and pyruvate (3-methyl-2-oxovalerate) in SW93 kernels. In addition, the higher α-ketoglutarate and lower levels of α-ketoglutarate derived amino acids and/or derivatives (glutamate, arginine and *N*-monomethylarginine) indicated that α-ketoglutarate was not the entry metabolite from carbon flux to nitrogen flux in SW93 kernels. The higher α-ketoglutarate in SW93 kernels reflected actually the consequence of the post-transcriptionally activated phenylalanine pathway via chorismate mutase, prephenate dehydratase, and phenylpyruvate aminotransferase. This metabolic shift was also observed in the coloration process of developing wild type tomato fruits^49^,^50^. This similarity suggests that it is interesting to investigate a broader range of plant systems in order to understand better the interface between primary and secondary metabolism and its impact on coloration.

### The Regulatory Mechanisms Underlying the Pigmentation in SW93 Kernel

The majority of maize varieties carry all the biosynthetic genes of pigmentation and the difference between “colored” and “uncolored” cultivars depends on the presence of “strong alleles” of the dominant regulatory genes that up-regulate the corresponding biosynthetic genes and determine the pigmentation of specific tissues^21^. As reviewed recently, these transcription factors usually function via the formation of various complexes^51^. Our transcriptomic data obtained here demonstrated that although all biosynthetic genes involved in anthocyanin and phlobaphene pathways were detected in both cultivars, the developmental stage dependent activation of these genes was only observed in SW93 (Fig. 5). Given that the development dependent activation of the flavonoid pathway was coincident only with the activation of two anthocyanin regulators (R1 and C1), the accumulation of anthocyanin in SW93 kernels seems highly likely to be the consequence of the activation of co-existing alleles of R1 and C1, two known developmental stimuli responsive anthocyanin transcription factors^21^,^28^. However, the kinetics of the third anthocyanin regulator PAC1 along the kernel development was quite similar in both cultivars, therefore, the exact details of the functional complex among C1, R1 and PAC1 remains unknown.

The formation of phlobaphene pigments in maize is developmentally controlled by the allelic variance in P1, which results in four phenotypes, P1-rr (red pericarp and red cob), P1-wr (white pericarp and red cob) P1-rw (red pericarp and white cob), and P1-ww (white pericarp and white cob)^27^,^52^. In a recent report, the coloration of an ancient Italy maize “Nero Spinoso” (P1-rw) was characterized to be under the control of a monogenic dominant gene, a strong allele of P1. Although SW93 looked phenotypically similar to P1-rw (Fig. 1A), the genetic evidence (Supplementary Fig. S1) indicated that the pigmentation was not under the control of a monogenic dominant gene. This fact aside, it was highly surprising that the expression of P1 was not detectable in the developing kernels of either cultivars, rendering the regulation of phlobaphene pigmentation in SW93 rather puzzling. One possible explanation is that instead of P1 other regulators of phlobaphene pigmentation exist in SW93 kernels, which are developmentally regulated in a manner similar to that of R1. Our qRT-PCR result excluded the possibility of P2 as such an alternative regulator, because the expression of neither P1 nor P2 was detected (Fig. 5C,D). Perhaps the two transcription factors showing similar expression patterns to that of R1 across kernel development are possible candidate regulators, however, the conclusive identification of alternative regulators rests on the further characterization of them (Supplementary Fig. S4). An additional explanation is the likely epigenetic regulation of P1 as reported previously^38^, which merits further investigations into the possible existence of tissue-specific regulatory elements within the P1 non-coding sequences or flanking regions, as well as DNA methylation. Given that we could not detect P1 expression across kernel development, we favor the first explanation. Nevertheless, the regulatory mechanisms underlying the pigmentation in SW93 maize kernel provided us a good chance to explore the molecular aspects of pigmentation in developing kernels using systems biology approaches.

In summary, taking the advantages of systems biology approach, we were able to unravel the transcriptomic and metabolomic kinetics of maize kernels across the development in general and characterize factors underlying the metabolic shifts in developing kernels of colored maize in particular. The coordinated metabolic shift between primary and secondary metabolism identified in colored SW93 maize will facilitate the further efforts to understand the complex relationships between crops rich in pigments, foods, and human health, and to improve the quality of crop-based foods via metabolic engineering.

## Methods

### Maize Materials

The two maize cultivars, SW48 (white kernels and white cobs) and SW93 (purple kernels and white cobs) were planted in the experimental fields of Shanghai Academy of Agricultural Science (Zhuanghang, Shanghai) during the summer season in 2014. These two inbred lines were used as male and female parent, respectively, to produce the popular fresh consumed sticky hybrid maize Huwucaihuanuo 1 (Supplementary Fig. S12). Maize kernels were collected at 11, 16 and 21 DAP, immediately frozen with liquid nitrogen, grounded into fine power in liquid nitrogen, aliquoted and stored at −80 °C prior to transcriptomic and metabolome analyses. Kernels taken from an individual corn cob were considered as one biological replicate, and both transcriptomic and metabolome analyses were performed with three replicates.

### Analysis of Total Anthocyanin Content

Anthocyanins were extracted as described previously by Žilić *et al.*^26^ with slight modifications. 120 mg fresh seed powder was extracted by mixing with 1 ml of methanol acidified with 1N HCl (85:15, v/v) and shaking for 30 min at room temperature. The crude extract was centrifuged at 12,000 rpm for 10 min, and 1 ml of the supernatant was mixed with 1 ml extraction solution. Then, the absorbance (A) of samples were measured at 535 and 700 nm, respectively, and concentrations (relative value) were calculated using the following equation: *A* = (*A*_*535*_−*A*_*700*_/3)^23^. The assay was performed in triplicate.

### Analysis of Flavan-4-ol Content

Flavan-4-ols were extracted as previously described by Robbins *et al.*^53^ with minor modifications. 150 mg fresh seed powder was incubated with 500 μl of butanol acidified with concentrated HCl (70:30, v/v) at 37 °C for 1 hour. The crude extract was centrifuged at 12,000 rpm for 10 min, and the supernatant was mixed with 1.5 ml extraction solution. The absorbance of samples were recorded at 550 nm and the relative abundance of flavan-4-ols was represented with *A*_*550*_. The assay was performed in triplicate.

### RNA Extraction, cDNA Library Construction and RNA Sequencing

Total RNA of each sample was extracted with the Qiagen Rneasy Mini Kit and digested with Dnase I according to the manufacturer’s instructions (Qiagen, Hilden, Germany). The integrity and size distribution of the RNA were verified with Agilent 2200 Bioanalyser (Agilent Technologies., USA.). Samples with the RNA Integrity Number ≥8.0 were used for cDNA library construction. The cDNA libraries for single-ending sequencing were constructed using Ion Total RNA-Seq Kit v2.0 (Life Technologies, Thermo Fisher Scientific, USA) according to the manufacturer’s instructions. The cDNA libraries were then processed for the Proton Sequencing process according to the commercially available protocols. Samples were diluted and mixed, and the mixture was processed on a OneTouch 2 instrument (Life Technologies) and enriched on a OneTouch 2 ES station (Life Technologies) for preparing the template-positive Ion PI™ Ion Sphere™ Particles (Life Technologies) according to Ion PI™ Template OT2 200 Kit v2.0 (Life Technologies). After enrichment, the mixed template-positive Ion PI™ Ion Sphere™ Particles of samples was loaded on to 1 P1v2 Proton Chip (Life Technologies) and sequenced on Proton Sequencers according to Ion PI Sequencing 200 Kit v2.0 (Life Technologies).

### RNA Sequencing Mapping

Before read mapping, clean reads were obtained from the raw reads by removing the adaptor sequences, reads with more than 5% ambiguous bases (noted as N), and low-quality reads containing more than 20 percent of bases with qualities less than 13. The clean reads were then aligned to maize genome (version: B73) using the MapSplice program (v2.1.8). In alignment, preliminary experiments were performed to optimize the alignment parameters *(-s 22 -p 12 -ins 6–del 6 –non-canonical*) to provide the largest information on the AS events^54^.

### Metabolite Profiling

Methanol extracts from 40 mg maize kernel powder per sample were analyzed with a metabolomic platform that combines UHPLC-MS/MS with GC-MS^55^,^56^,^57^. Metabolite identification was performed as reported previously^20^,^58^ based on the comparison to an in-house library entries of purified metabolite standards, containing the retention index (RI), mass to charge ratio (m/z), and MS/MS spectral data.

### Statistical Analysis

Differentially expressed genes were filtered by applying DEseq algorithm^59^ with Fold Change >2 or <0.5, and false discovery rate (FDR) <0.05^60^. Gene ontology (GO) analysis was performed to facilitate elucidating the biological implications of unique genes in the significant or representative profiles of the differentially expressed gene in the experiment^61^. GO annotations were downloaded from NCBI (http://www.ncbi.nlm.nih.gov/), UniProt (http://www.uniprot.org/) and the Gene Ontology (http://www.geneontology.org/). Fisher’s exact test was applied to identify the significant GO categories and pathway with FDR <0.05^62^. Metabolite abundances were quantified using peak areas. The missing values of a giving metabolite were inputted with the detected minimum value for statistical analysis, assuming that they were below the limits of instrument detection sensitivity. Metabolitc differences between different groups were compared by using Welch’s two-sample t-test and significant level was declared for p-values less than 0.05.

Principle Component Analysis was performed with SIMCA-P software and “Par” was selected for data scaling type. Two-way ANOVA and ASCA (ANOVA-Simultaneous Component Analysis) were performed with MetATT^63^ (http://www.metaboanalyst.ca/) using “Pareto Scaling” for data normalization. Two-way ANOVA type used is “within subjects ANOVA”, significance threshold is defined as the corrected p-value < 0.05 and False Discovery Rate <0.05 was chosen for multiple testing correction. ASCA was performed with default parameters supplied by the website. Hierarchical clustering of metabolites and enzyme coding genes (ECG) was performed with MultiExperiment Viewer (MeV) version 4.8.

## Additional Information

**How to cite this article**: Hu, C. *et al.* Characterization of factors underlying the metabolic shifts in developing kernels of colored maize. *Sci. Rep.* **6**, 35479; doi: 10.1038/srep35479 (2016).

## Supplementary Material

Supplementary Information

Supplementary Tables
