# Supplementary material for: Characterization of factors underlying the metabolic shifts in developing kernels of colored maize
Source: Sci Rep. 2016 Oct 14;6:35479. doi: 10.1038/srep35479 (PMC5064397; doi:10.1038/srep35479)
Supplement: Supplementary Information [file srep35479-s1.pdf]

Characterization of factors underlying the metabolic shifts in developing kernels of colored maize

Chaoyang Hu, Quanlin Li, Xuefang Shen, Sheng Quan, Hong Lin, Lei Duan, Yifa Wang, Qian Luo, Guorun Qu, Qing Han, Yuan Lu, Dabing Zhang, Zheng Yuan and Jianxin Shi

Supplementary Information

Supplementary Figures

Supplemental Figure 1. **Principle component analysis of enzyme coding genes**. Red, green and blue colors represent samples at I, II and III, respectively. Box and triangle denote samples of SW93 and SW48, respectively. I, II and III refer to 11, 16 and 21 DAP, respectively.

Supplemental Figure 2. **Scree plots for each models with enzyme coding genes**.

Supplemental Figure 3. **Scree plots for each models with metabolites**.

Supplemental Figure 4. **Hierarchical clustering analysis showing identical expression patterns of two transcription factors to that of *R1***.

Supplemental Figure 5. **Integration of metabolite and gene expression changes of glycolysis pathway**. (A) Kinetics of glycolytic metabolites along the development. *denotes the metabolite level at II or III was significantly different from that at I of the same cultivar. Black and purple stars denote SW48 and SW93, respectively. (B) Schematic maps of glycolysis. 2.7.1.1, hexokinase; 5.3.1.9, gluocose-phosphate isomerase; 2.7.1.11, 6-phosphofructokinase; 3.1.3.11, fructose-bisphosphatase; 4.1.2.13, fructose-bisphosphate aldolase; 1.2.1.12, glyceraldehyde-3-phosphate dehydrogenase; 2.7.2.3, phosphoglycerate kinase; 1.2.1.9, glyceraldehyde-3-phosphate dehydrogenase; 5.4.2.11, 5.4.2.12, phosphoglycerate mutase; 4.2.1.11, phosphopyruvate hydratase; 2.7.1.40, pyruvate kinase. (C) Expression patterns of genes involved in glycolysis. The expression profiles of the corresponding genes were presented in Supplemental Table 8.

Supplemental Figure 6. **Integration of metabolite and gene expression changes of TCA cycle**. (A) Kinetics of TCA cycle metabolites along the development. *denotes

the metabolite level at II or III was significantly different from that at I of the same cultivar. Black and purple stars denote SW48 and SW93, respectively. (B) Schematic maps of TCA cycle. 4.2.1.3, aconitate hydratase; 1.1.1.41, isocitrate dehydrogenase (NAD+); 1.1.1.42, isocitrate dehydrogenase (NADP+); 1.2.4.2, oxoglutarate dehydrogenase; 2.3.1.61, dihydrolipoyllysine-residue succinyltransferase; 6.2.1.4, succinate-CoA ligase (GDP-forming); 1.3.5.1, succinate dehydrogenase; 4.2.1.2, fumarate hydratase; 1.1.1.37, malate dehydrogenase; 2.3.3.1, citrate (Si)-synthase; 2.3.3.8, ATP citrate synthase. (C) Expression patterns of genes involved in TCA cycle. The expression profiles of the corresponding genes were presented in Supplemental Table 8.

Supplemental Figure 7. **Integration of metabolite and gene expression changes of pentose phosphate pathway (PPP)**. (A) Kinetics of PPP metabolites along the development. *denotes the metabolite level at II or III was significantly different from that at I of the same cultivar. Black and purple stars denote SW48 and SW93, respectively. (B) Schematic maps of PPP. 1.1.1.49, glucose-6-phosphate dehydrogenase (NADP+); 3.1.1.31, 6-phosphogluconolactonase; 1.1.1.44, phosphogluconic acid dehydrogenase; 5.1.3.1, ribulose-phosphate 3-epimerase; 5.3.1.6, ribose-5-phosphate isomerase; 2.2.1.1 and 2.2.1.2, transketolase. (C) Expression patterns of genes involved in PPP. The expression profiles of the corresponding genes were presented in Supplemental Table 8.

Supplemental Figure 8. **Integration of metabolite and gene expression changes of purine metabolism**. (A) Kinetics of purine metabolism metabolites along the development. *denotes the metabolite level at II or III was significantly different from that at I of the same cultivar. Black and purple stars denote SW48 and SW93, respectively. (B) Schematic maps of purine metabolism. 2.4.2.7, adenine phosphoribosyltransferase; 3.1.3.5, 5'-nucleotidase; 3.5.4.4, adenosine deaminase; 3.5.4.6, AMP deaminase; 2.4.2.8, hypoxanthine phosphoribosyltransferase; 1.1.1.205, IMP dehydrogenase; 6.3.5.2, GMP synthase; 1.7.3.3, uric acid oxidase. (C) Expression patterns of genes involved in purine metabolism. The expression profiles of the corresponding genes were presented in Supplemental Table 8.

Supplemental Figure 9. **Integration of metabolite and gene expression changes of serine and glycine metabolism**. (A) Kinetics of serine and glycine metabolism metabolites along the development. *denotes the metabolite level at II or III was significantly different from that at I of the same cultivar. Black and purple stars denote SW48 and SW93, respectively. (B) Schematic maps of serine and glycine metabolism. 2.3.1.30, serine O-acetyltransferase; 4.2.1.20, tryptophan synthase; 2.1.2.1, glycine hydroxymethyltransferase; 2.5.1.47, cysteine synthase; 4.1.2.5, threonine aldolase; 4.4.1.8, cystathionine beta-lyase; 4.2.3.1, threonine synthase; 2.1.1.10, homocysteine S-methyltransferase; 2.1.1.14, homocysteine methylase; 2.7.1.39, homoserine kinase; 2.5.1.6, methionine adenosyltransferase; 2.1.1.37, DNA (cytosine-5-)-methyltransferase; 3.3.1.1, adenosylhomocysteinase. (C) Expression patterns of genes involved in serine and glycine metabolism. The expression profiles of the corresponding genes were presented in Supplemental Table 8.

Supplemental Figure 10. **Integration of metabolite and gene expression changes of nicotinamide metabolism**. (A) Kinetics of nicotinamide metabolism metabolites along the development. *denotes the metabolite level at II or III was significantly different from that at I of the same cultivar. Black and purple stars denote SW48 and SW93, respectively. (B) Schematic maps of nicotinamide metabolism. 6.3.5.1, NAD+ synthase; 2.7.7.1, nicotinamide-nucleotide adenylyltransferase; 3.1.3.5, 5'-nucleotidase; 3.2.2.3, uridine nucleosidase; 6.3.4.21, nicotinate phosphoribosyltransferase. (C) Expression patterns of genes involved in nicotinamide metabolism. The expression profiles of the corresponding genes were presented in Supplemental Table 8.

Supplemental Figure 11. **Integration of metabolite and gene expression changes of shikimate pathway**. (A) Kinetics of shikimate pathway metabolites along the development. *denotes the metabolite level at II or III was significantly different from that at I of the same cultivar. Black and purple stars denote SW48 and SW93, respectively. (B) Schematic maps of shikimate pathway. 2.5.1.54, 3-deoxy-7-phosphoheptulonate synthase; 4.2.3.4, 3-dehydroquinate synthase; 4.2.1.10, 3-dehydroquinate dehydratase; 1.1.1.25, shikimate dehydrogenase; 2.7.1.71, shikimate kinase; 2.5.1.19, 3-phosphoshikimate 1-carboxyvinyltransferase; 4.2.3.5, chorismate

synthase; 4.1.3.27, anthranilate synthase; 2.4.2.18, anthranilate phosphoribosyltransferase; 5.3.1.24, phosphoribosylanthranilate isomerase; 4.1.1.48, indole-3-glycerol-phosphate synthase; 4.2.1.20, tryptophan synthase; 5.4.99.5, chorismate mutase; 2.6.1.79, glutamate-prephenate aminotransferase; 1.3.1.78, arogenate dehydrogenase (NADP+); 4.2.1.91, arogenate dehydratase; 2.6.1.5, yrosine transaminase. (C) Expression patterns of genes involved in shikimate pathway. The expression profiles of the corresponding genes were presented in Supplemental Table 8.

Supplemental Figure 12. **Genetic analysis of SW48 and SW93 maize and their offspring progenies**.

Legends of Supplemental Tables

Supplemental Table 1. Raw data of RNA-Seq.

Supplemental Table 2. Heat map of statistically significant metabolites profiled in this study. Red and green shaded cells indicate p-value ≤ 0.05 (red indicates that the mean values are significantly higher for that comparison; green values significantly lower).

Supplemental Table 3. The list of enzyme coding genes that were affected by time, cultivar or their interactions. Yellow color indicates the expression level of these genes were simultaneously affected by time, cultivar and their interaction.

Supplemental Table 4. The list of metabolites that were affected by time, cultivar or their interactions. Yellow color indicates the abundance of these metabolites were simultaneously affected by time, cultivar and their interaction.

Supplemental Table 5. List of well-modeled genes associtated with time, cultivar and interaction, respectively.

Supplemental Table 6. List of well-modeled metabolites associtated with time, cultivar and interaction, respectively.

Supplemental Table 7. Significantly enriched GO terms among genes differently expressed between SW93 and SW48 at three time points.

Supplemental Table 8.Comparision of expression levels of enzyme coding genes between different maize kernel samples.

Supplemental Table 9.Heat map of statistically significant metabolites profiled in this study. Red and green shaded cells indicate p-value ≤ 0.05 (red indicates that the mean values are significantly higher for that comparison; green values significantly lower).

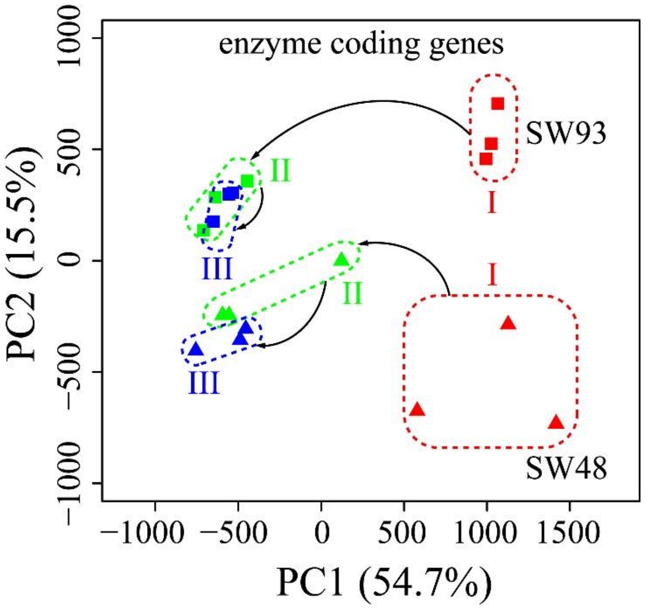

Supplementary Figure S1 | Principle component analysis of enzyme coding genes. Red, green and blue colors represent samples at I, II and III, respectively. Box and triangle denote samples of SW93 and SW48, respectively. I, II and III refer to 11, 16 and 21 DAP, respectively.

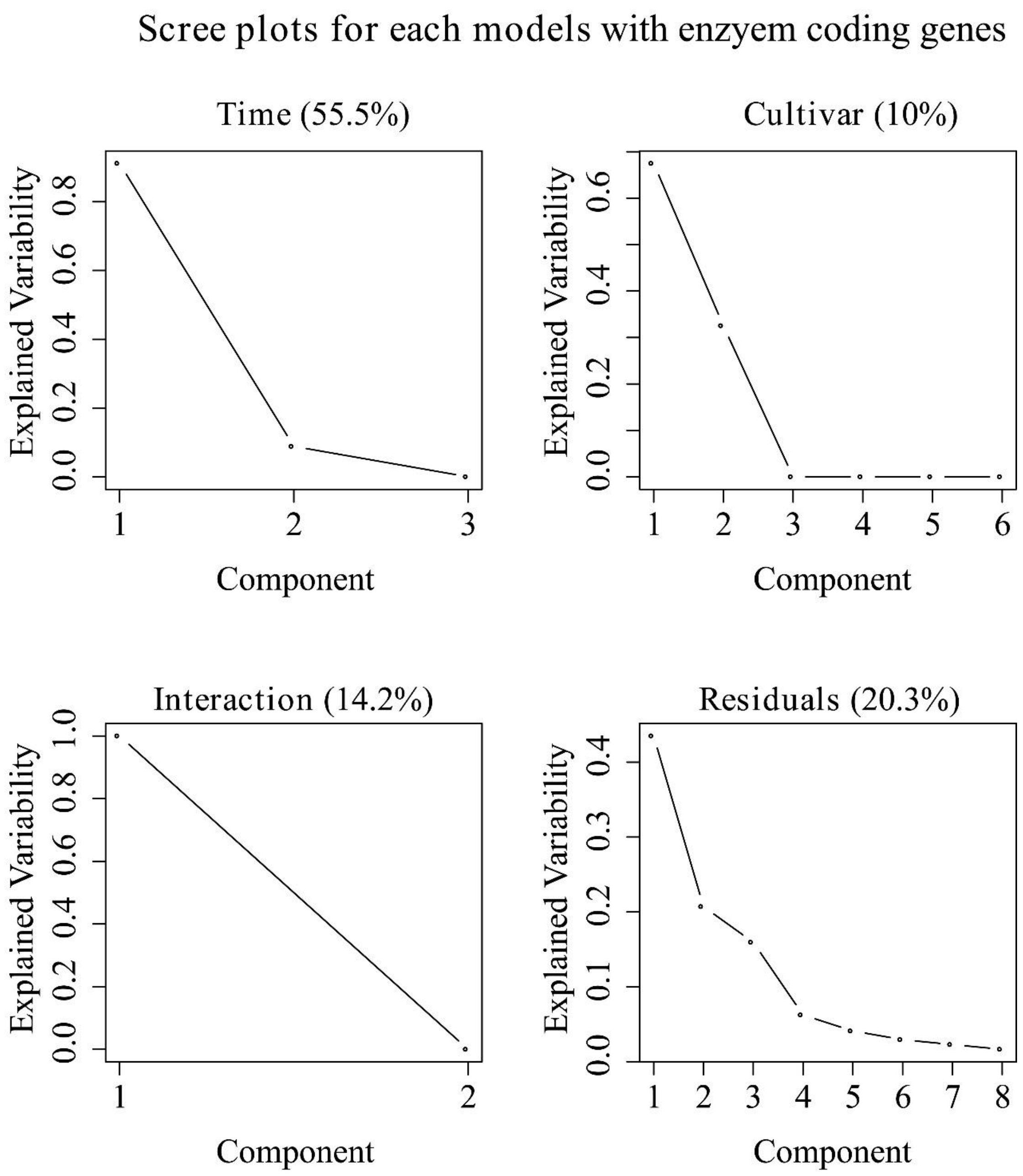

Supplementary Figure S2 | Scree plots for each models with enzyme coding genes.

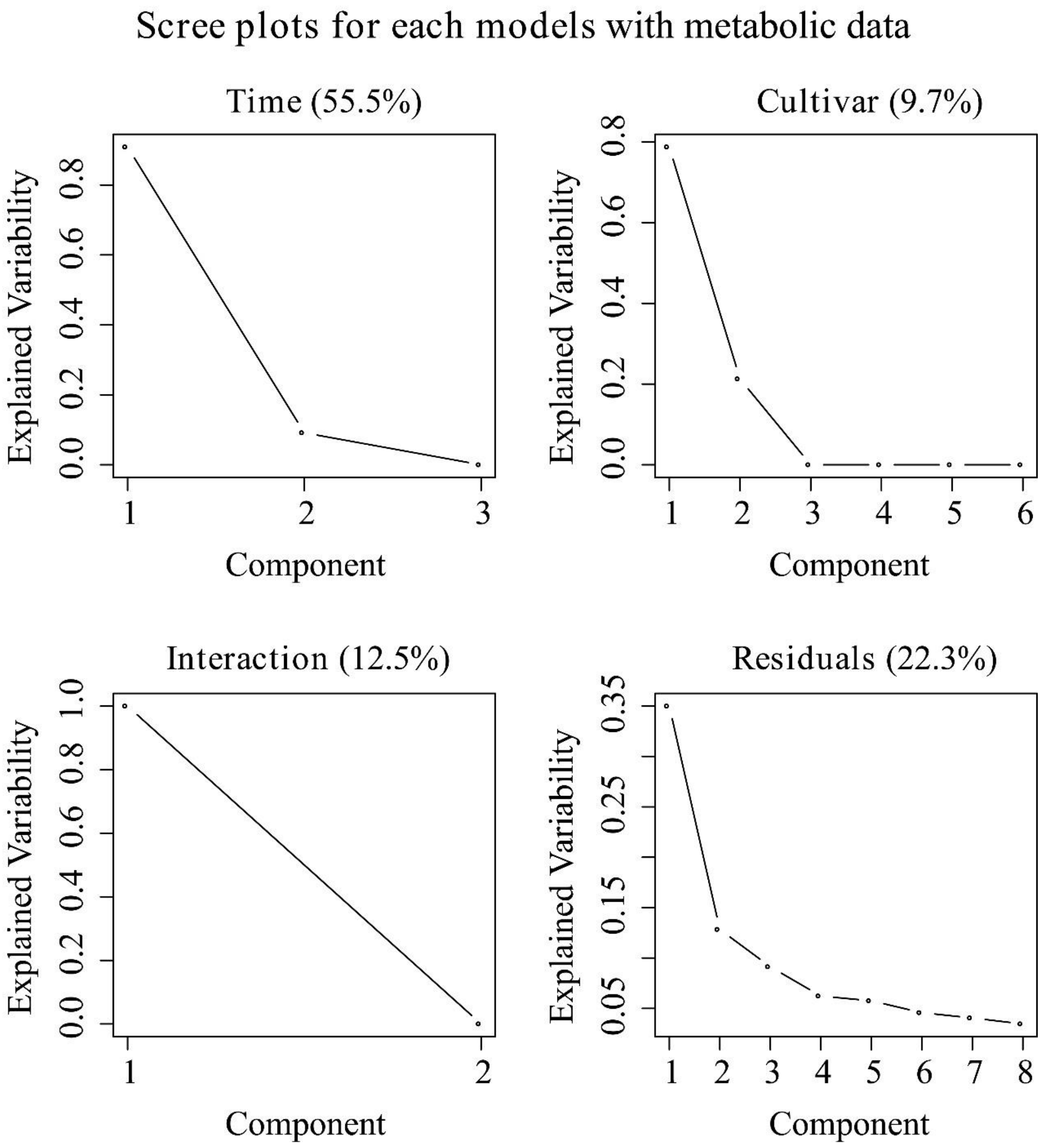

Supplementary Figure S3 | Scree plots for each models with metabolites.

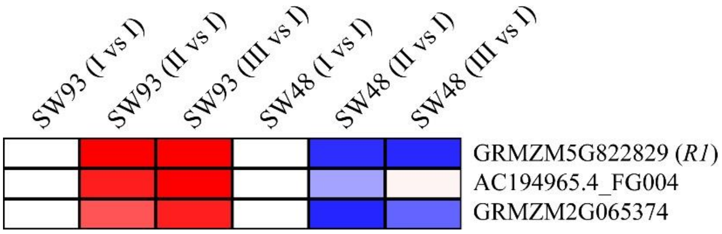

Supplementary Figure S4 | Hierarchical clustering analysis showing identical expression patterns of two transcription factors to that of *R1*.

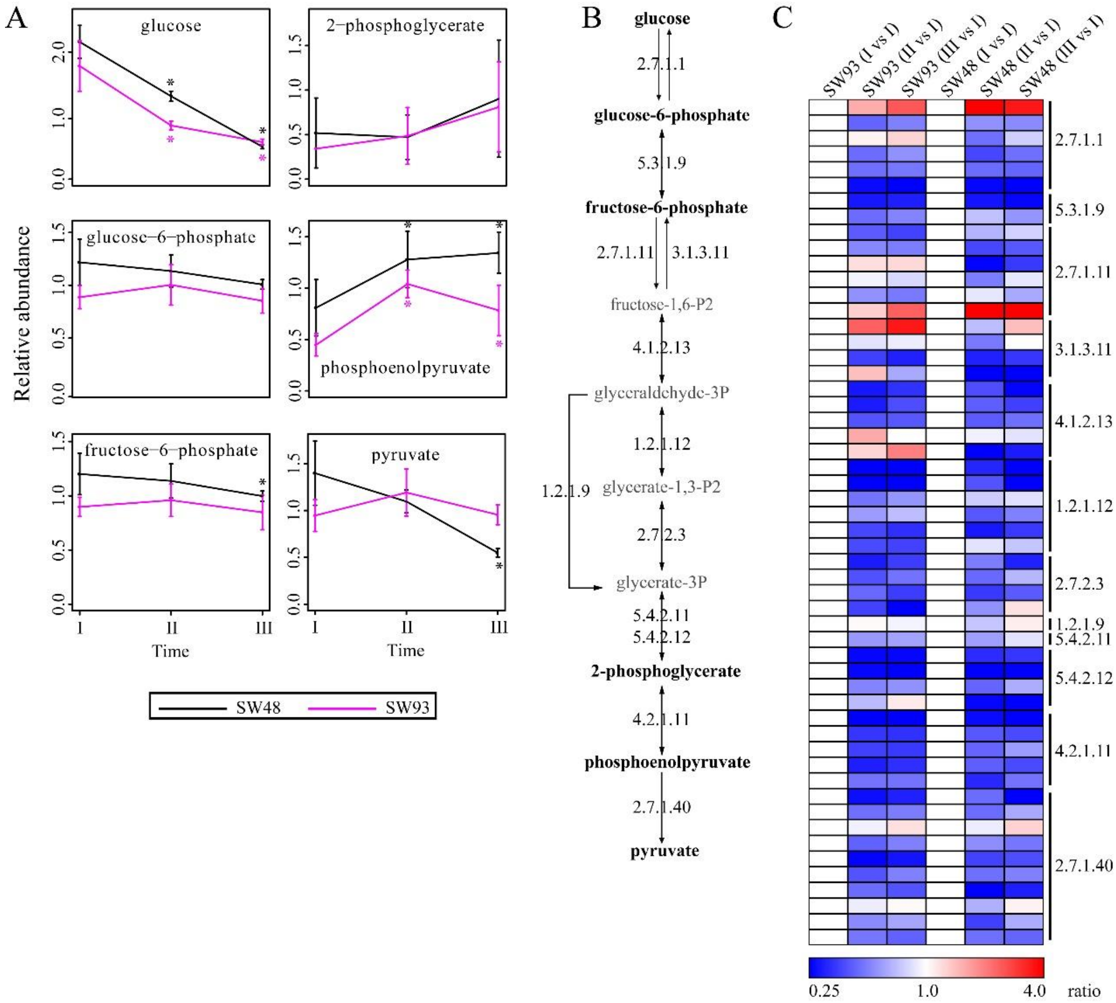

Supplementary Figure S5 | Integration of metabolite and gene expression changes of glycolysis pathway. A, Kinetics of glycolytic metabolites along the development. *denotes the metabolite level at II or III was significantly different from that at I of the same cultivar. Black and purple stars denote SW48 and SW93, respectively. B, Schematic maps of glycolysis. Enzymes are shown as EC code numbers: EC: 2.7.1.1, hexokinase; EC: 5.3.1.9, gluocose-phosphate isomerase; EC: 2.7.1.11, 6-phosphofructokinase; EC: 3.1.3.11, fructose-bisphosphatase; EC: 4.1.2.13, fructose-bisphosphate aldolase; EC: 1.2.1.12, glyceraldehyde-3-phosphate dehydrogenase; EC: 2.7.2.3, phosphoglycerate kinase; EC: 1.2.1.9, glyceraldehyde-3-phosphate dehydrogenase; EC: 5.4.2.11 and EC: 5.4.2.12, phosphoglycerate mutase; EC: 4.2.1.11, phosphopyruvate hydratase; EC: 2.7.1.40, pyruvate kinase. C, Expression patterns of genes involved in glycolysis. The expression profiles of the corresponding genes were presented in Supplementary Table S8.

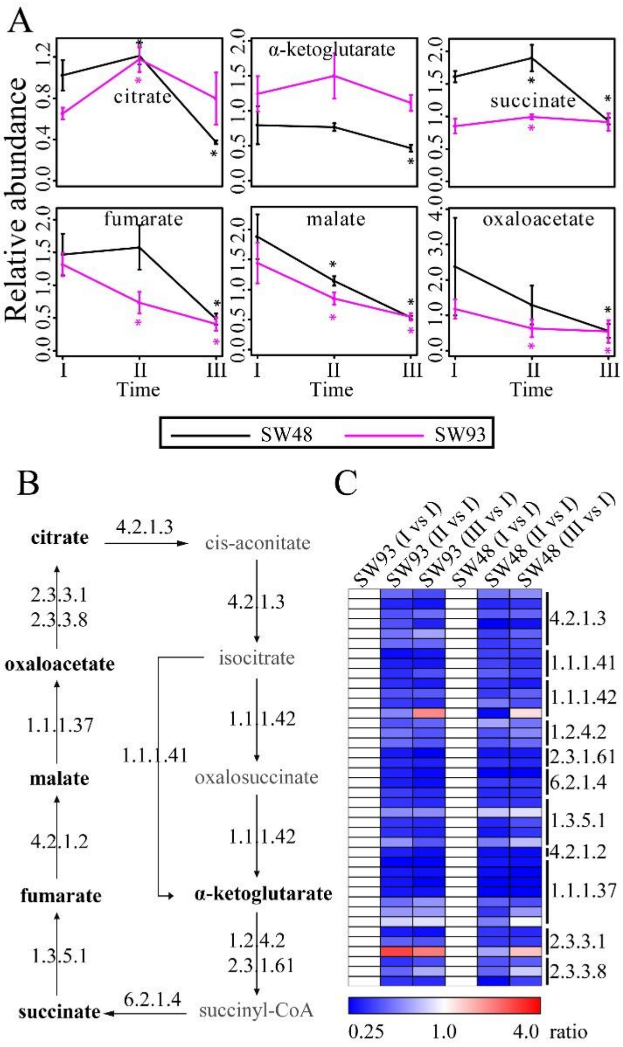

Supplementary Figure S6 | Integration of metabolite and gene expression changes of TCA cycle. A, Kinetics of TCA cycle metabolites along the development. *denotes the metabolite level at II or III was significantly different from that at I of the same cultivar. Black and purple stars denote SW48 and SW93, respectively. B, Schematic maps of TCA cycle. Enzymes are shown as EC code numbers: EC: 4.2.1.3, aconitate hydratase; EC: 1.1.1.41, isocitrate dehydrogenase (NAD+); EC: 1.1.1.42, isocitrate dehydrogenase (NADP+); EC: 1.2.4.2, oxoglutarate dehydrogenase; EC: 2.3.1.61, dihydrolipoyllysine-residue succinyltransferase; EC: 6.2.1.4, succinate-CoA ligase (GDP-forming); EC: 1.3.5.1, succinate dehydrogenase; EC: 4.2.1.2, fumarate hydratase; EC: 1.1.1.37, malate dehydrogenase; EC: 2.3.3.1, citrate (Si)-synthase; EC: 2.3.3.8, ATP citrate synthase. C, Expression patterns of genes involved in TCA cycle. The expression profiles of the corresponding genes were presented in Supplementary Table S8.

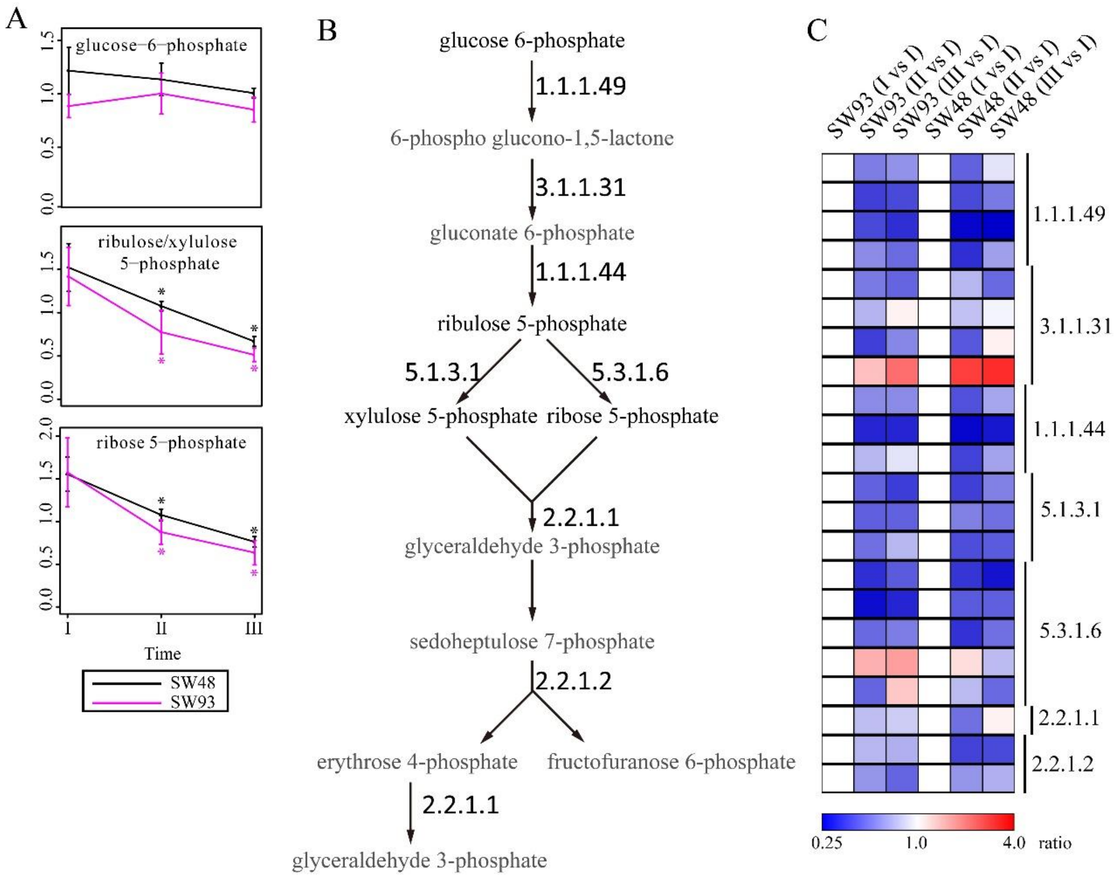

Supplementary Figure S7 | Integration of metabolite and gene expression changes of pentose phosphate pathway (PPP). A, Kinetics of PPP metabolites along the development. *denotes the metabolite level at II or III was significantly different from that at I of the same cultivar. Black and purple stars denote SW48 and SW93, respectively. B, Schematic maps of PPP. Enzymes are shown as EC code numbers: EC: 1.1.1.49, glucose-6-phosphate dehydrogenase (NADP+); EC: 3.1.1.31, 6-phosphogluconolactonase; EC: 1.1.1.44, phosphogluconic acid dehydrogenase; EC: 5.1.3.1, ribulose-phosphate 3-epimerase; EC: 5.3.1.6, ribose-5-phosphate isomerase; EC: 2.2.1.1 and EC: 2.2.1.2, transketolase. C, Expression patterns of genes involved in PPP. The expression profiles of the corresponding genes were presented in Supplementary Table S8.

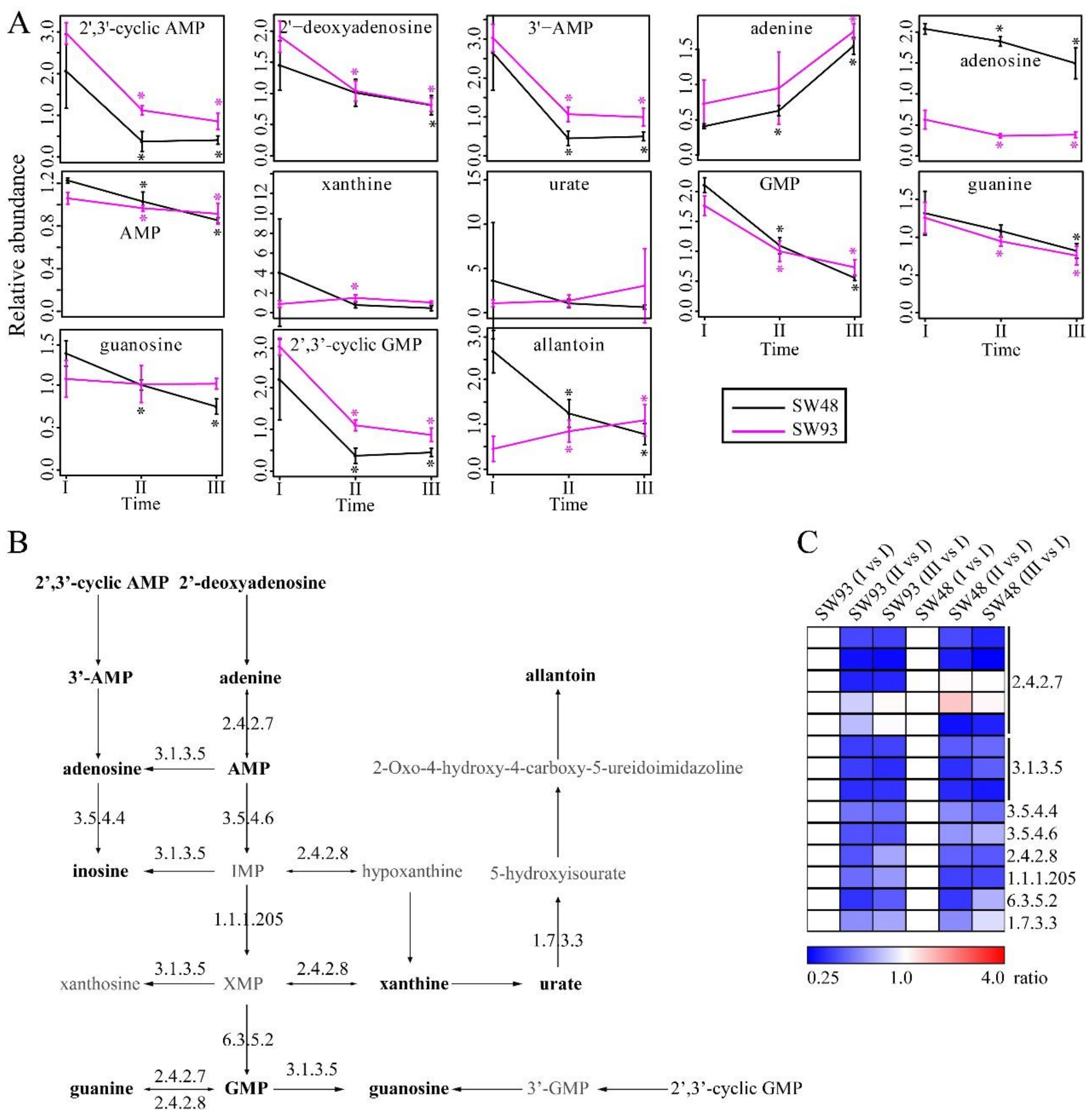

Supplementary Figure S8 | Integration of metabolite and gene expression changes of purine metabolism. A, Kinetics of purine metabolism metabolites along the development. *denotes the metabolite level at II or III was significantly different from that at I of the same cultivar. Black and purple stars denote SW48 and SW93, respectively. B, Schematic maps of purine metabolism. Enzymes are shown as EC code numbers: EC: 2.4.2.7, adenine phosphoribosyltransferase; EC: 3.1.3.5, 5'-nucleotidase; EC: 3.5.4.4, adenosine deaminase; EC: 3.5.4.6, AMP deaminase; EC: 2.4.2.8, hypoxanthine phosphoribosyltransferase; EC: 1.1.1.205, IMP dehydrogenase; EC: 6.3.5.2, GMP synthase; EC: 1.7.3.3, uric acid oxidase. C, Expression patterns of genes involved in purine metabolism. The expression profiles of the corresponding genes were presented in Supplementary Table S8.

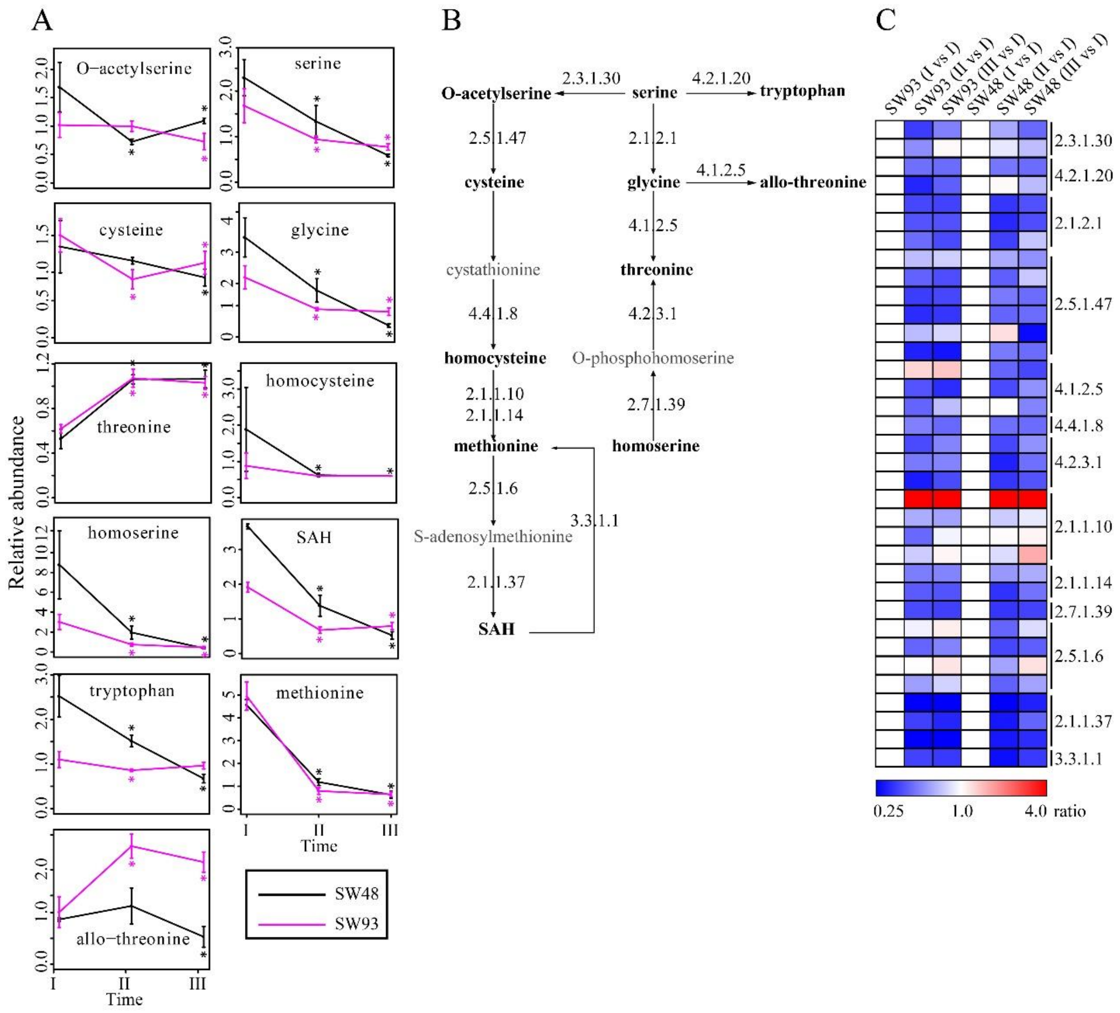

Supplementary Figure S9 | Integration of metabolite and gene expression changes of serine and glycine metabolism. A, Kinetics of serine and glycine metabolism metabolites along the development. *denotes the metabolite level at II or III was significantly different from that at I of the same cultivar. Black and purple stars denote SW48 and SW93, respectively. B, Schematic maps of serine and glycine metabolism. Enzymes are shown as EC code numbers: EC: 2.3.1.30, serine O-acetyltransferase; EC: 4.2.1.20, tryptophan synthase; EC: 2.1.2.1, glycine hydroxymethyltransferase; EC: 2.5.1.47, cysteine synthase; EC: 4.1.2.5, threonine aldolase; EC: 4.4.1.8, cystathionine beta-lyase; EC: 4.2.3.1, threonine synthase; EC: 2.1.1.10, homocysteine S-methyltransferase; EC: 2.1.1.14, homocysteine methylase; EC: 2.7.1.39, homoserine kinase; EC: 2.5.1.6, methionine adenosyltransferase; EC: 2.1.1.37, DNA (cytosine-5-)-methyltransferase; EC: 3.3.1.1, adenosylhomocysteinase. C, Expression patterns of genes involved in serine and glycine metabolism. The expression profiles of the corresponding genes were presented in Supplementary Table S8.

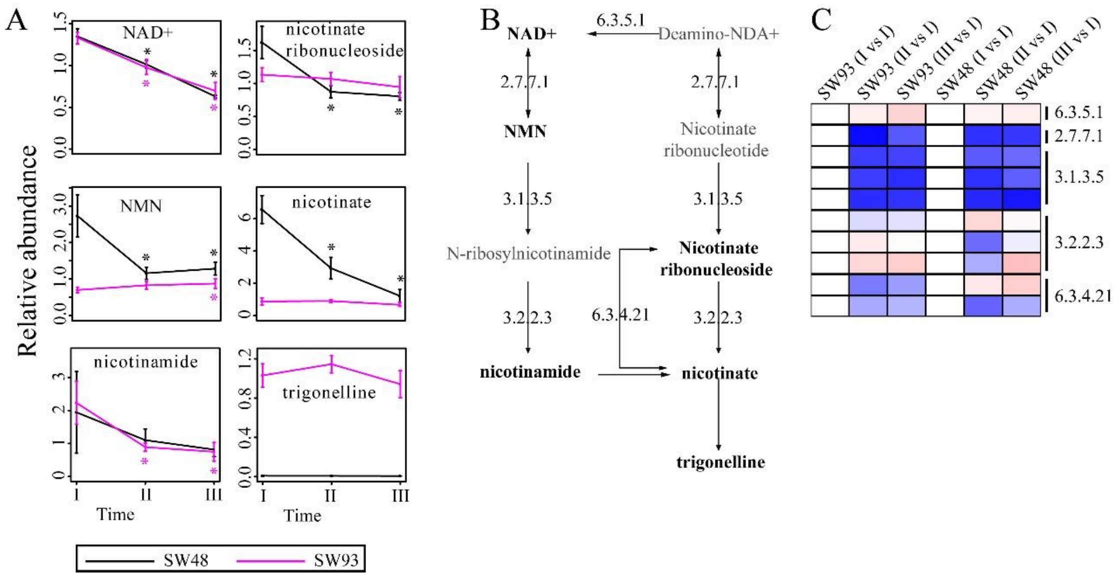

Supplementary Figure S10 | Integration of metabolite and gene expression changes of nicotinamide metabolism. A, Kinetics of nicotinamide metabolism metabolites along the development. *denotes the metabolite level at II or III was significantly different from that at I of the same cultivar. Black and purple stars denote SW48 and SW93, respectively. B, Schematic maps of nicotinamide metabolism. Enzymes are shown as EC code numbers: EC: 6.3.5.1, NAD+ synthase; EC: 2.7.7.1, nicotinamide-nucleotide adenylyltransferase; EC: 3.1.3.5, 5'-nucleotidase; EC: 3.2.2.3, uridine nucleosidase; EC: 6.3.4.21, nicotinate phosphoribosyltransferase. C, Expression patterns of genes involved in nicotinamide metabolism. The expression profiles of the corresponding genes were presented in Supplementary Table S8.

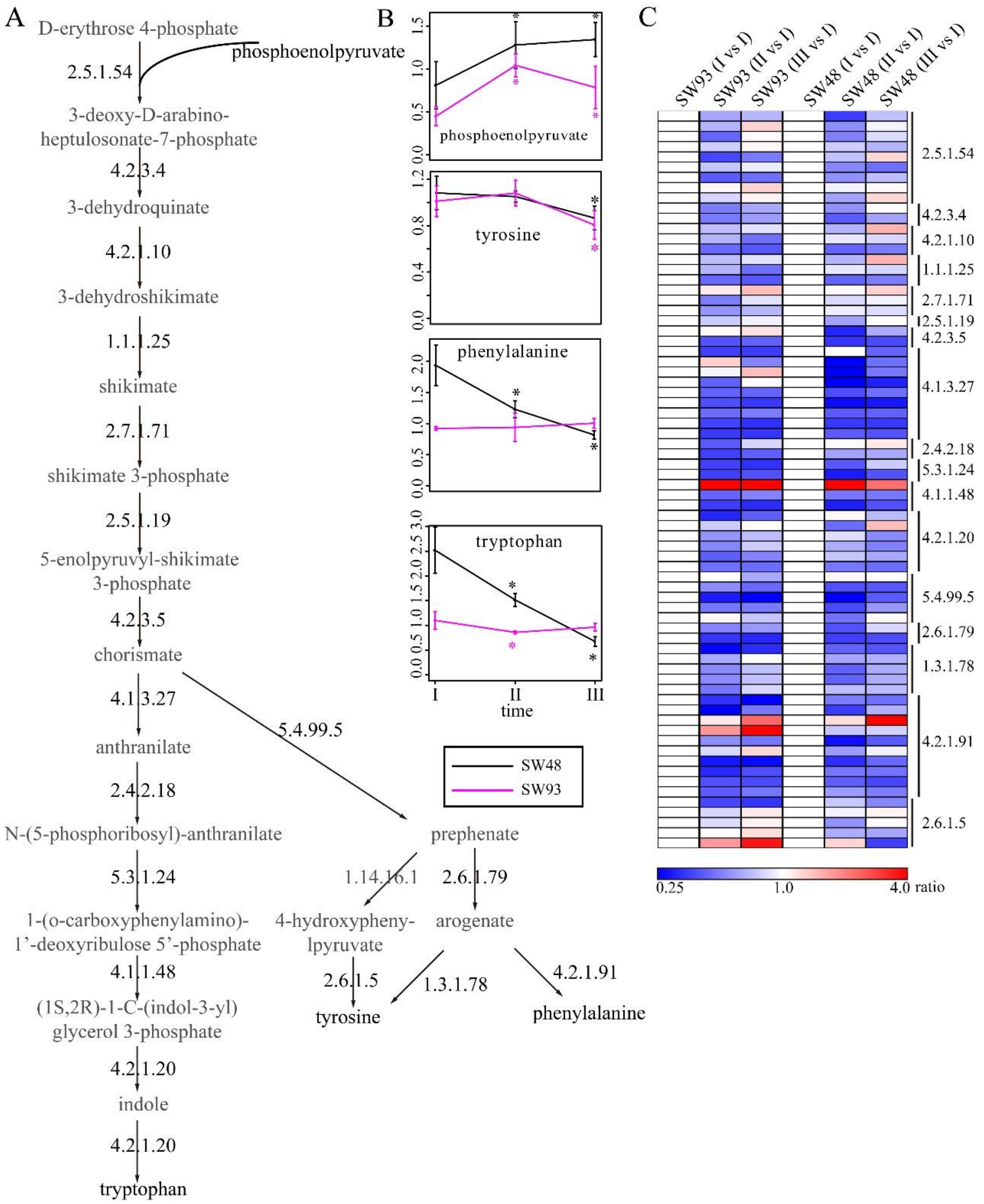

Supplementary Figure S11 | Integration of metabolite and gene expression changes of shikimate pathway. A, Kinetics of shikimate pathway metabolites along the development. *denotes the metabolite level at II or III was significantly different from that at I of the same cultivar. Black and purple stars denote SW48 and SW93, respectively. B, Schematic maps of shikimate pathway. Enzymes are shown as EC code numbers: EC: 2.5.1.54, 3-deoxy-7-phosphoheptulonate synthase; EC: 4.2.3.4, 3-dehydroquinate synthase; EC: 4.2.1.10, 3-dehydroquinate dehydratase; EC: 1.1.1.25, shikimate dehydrogenase; EC: 2.7.1.71, shikimate kinase; EC: 2.5.1.19, 3-phosphoshikimate 1-carboxyvinyltransferase; EC: 4.2.3.5, chorismate synthase; EC: 4.1.3.27, anthranilate synthase; EC:

2.4.2.18, anthranilate phosphoribosyltransferase; EC: 5.3.1.24, phosphoribosylanthranilate isomerase; EC: 4.1.1.48, indole-3-glycerol-phosphate synthase; EC: 4.2.1.20, tryptophan synthase; EC: 5.4.99.5, chorismate mutase; EC: 2.6.1.79, glutamate-prephenate aminotransferase; EC: 1.3.1.78, arogenate dehydrogenase (NADP+); EC: 4.2.1.91, arogenate dehydratase; EC: 2.6.1.5, yrosine transaminase. C, Expression patterns of genes involved in shikimate pathway. The expression profiles of the corresponding genes were presented in Supplementary Table S8.

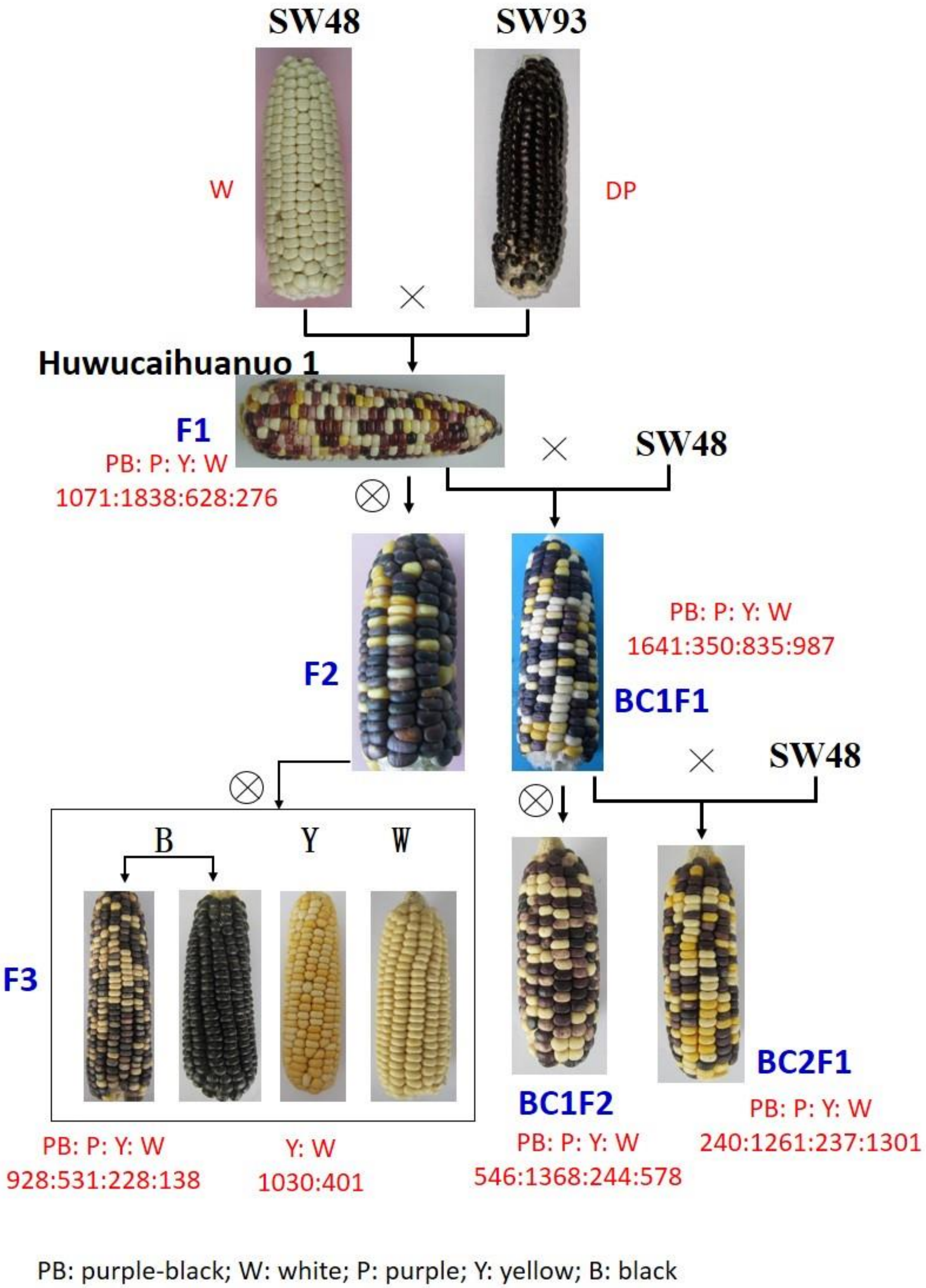

Supplementary Figure 12 | Genetic analysis of SW48 and SW93 maize and their offspring progenies.
